# Draft Genome Sequence of *Dietzia* sp. Strain UCD-THP (Phylum *Actinobacteria*)

**DOI:** 10.1128/genomeA.00197-13

**Published:** 2013-05-09

**Authors:** Amanda L. Diep, Jenna M. Lang, Aaron E. Darling, Jonathan A. Eisen, David A. Coil

**Affiliations:** University of California, Davis, Genome Center, Davis, California, USA

## Abstract

Here, we present the draft genome sequence of an actinobacterium, *Dietzia* sp. strain UCD-THP, isolated from a residential toilet handle. The assembly contains 3,915,613 bp. The genome sequences of only two other *Dietzia* species have been published, those of *Dietzia alimentaria* and *Dietzia cinnamea.*

## GENOME ANNOUNCEMENT

Members of the *Dietzia* genus have been isolated from diverse environments, including Korean food (1), a soda lake (2), and a swab sample from a human patient (3). *Dietzia* spp. are characterized as Gram positive and can be seen as both cocci and rods. Colonies usually appear orange to coral.

*Dietzia* sp. strain UCD-THP was isolated from a residential toilet handle in Davis, CA, as part of a project to produce built-environment reference genomes. Swabs were incubated overnight at 37°C in Luria broth (LB) and plated on LB agar, and colonies were isolated by serial dilution streaking. Single colonies were grown overnight in a mixture of 1% tryptone, 1% NaCl, 0.5% yeast extract, and 0.4% glucose, adjusted to pH 9. One isolate was identified by sequencing the 16S rRNA gene PCR product produced by the 1391R and 27F primers. Cells were lysed by bead beating and freeze-thawing. DNA was extracted with an equal volume of phenol-chloroform and subjected to ethanol precipitation.

Two Illumina paired-end libraries were generated using a TruSeq DNA sample prep v2 kit (Illumina) and a Nextera DNA sample prep kit (Illumina). Fragments of 300 to 600 bp were selected using a Pippin Prep (Sage Science). Libraries were sequenced on an Illumina MiSeq, with a read length of 250 bp, trimmed to 160 bp prior to assembly. This produced a total of 7,104,230 paired-end reads. Quality trimming and error correction of the reads resulted in 6,516,092 high-quality reads. These steps were performed using the A5 assembly pipeline (4). This pipeline automates data cleaning, error correction, contig assembly, and scaffolding. An additional assembly was generated using the CLC Genomics Workbench. The two assemblies were mapped to each other using progressiveMauve (5), and scaffolds from the CLC assembly that were not present in the A5 assembly were removed. The resulting consensus assembly had 141 scaffolds (minimum, 321 bp; maximum, 506,669 bp; *N*_50_, 157,523 bp). During scaffolding, some contigs were merged based on short overlaps and read-pair information, yielding a final collection of 219 contigs in 141 scaffolds that were submitted to GenBank. This final assembly had 3,915,613 bp, with a G+C content of 69.5% and a coverage estimate of 266×. Genome completeness was assessed using the PhyloSift software (A. Darling, G. Jospin, E. Lowe, E. Matsen, H. Bik, and J. Eisen, unpublished data), which searches for a list of 40 highly conserved single-copy marker genes (D. Wu, G. Jospin, and J. Eisen, unpublished data), of which all were found in this assembly.

Annotation was performed using the RAST server (6). *Dietzia* sp. strain UCD-THP contains 3,614 predicted protein-coding sequences and 50 predicted noncoding RNAs. A phylogenetic tree of all available cultured isolates of *Dietzia* was produced using the Ribosomal Database Project (RDP), which implements a weighted neighbor-joining algorithm (7). *Dietzia* sp. strain UCD-THP falls within a poorly resolved paraphyletic clade containing 7 species of *Dietzia* (http://dx.doi.org/10.6084/m9.figshare.646178). Because the 16S rRNA gene sequence of *Dietzia* sp. strain UCD-THP has >99% identity to homologs from several species of cultured isolates, and the phylogenetic relationships among those species are unclear, we have been unable to assign a species name to this isolate.

### Nucleotide sequence accession numbers.

This Whole-Genome Shotgun project has been deposited at DDBJ/EMBL/GenBank under the accession no. AOSR00000000. The version described in this paper is the first version, accession no. AOSR01000000. Illumina reads are available at http://dx.doi.org/10.6084/m9.figshare.644656.
